# Is crying a self-soothing behavior?

**DOI:** 10.3389/fpsyg.2014.00502

**Published:** 2014-05-28

**Authors:** Asmir Gračanin, Lauren M. Bylsma, Ad J. J. M. Vingerhoets

**Affiliations:** ^1^Department of Psychology, Faculty of Humanities and Social Sciences, University of RijekaRijeka, Croatia; ^2^Department of Psychiatry, University of PittsburghPittsburgh, PA, USA; ^3^Department of Medical and Clinical Psychology, Tilburg UniversityTilburg, Netherlands

**Keywords:** emotion regulation, self-soothing, social-soothing, mood management, crying

## Abstract

This contribution describes the current state-of-the-art of the scientific literature regarding the self-soothing effects of crying. Starting from the general hypothesis that crying is a self-soothing behavior, we consider different mechanisms through which these effects may appear. In the first section, we briefly explain the main functions of human crying. Then we define self-soothing in terms of homeostatic processes of mood regulation and stress reduction and we underline the importance of distinguishing self-soothing effects of crying from social-soothing that it may elicit. We then provide a comprehensive review of the putative mood-enhancing and -relieving effects of crying and their variations stemming from characteristics of crying person, antecedents, manifestations, and social consequences of crying. We also discuss the possible methodological explanations for the seemingly discrepant findings regarding mood improvement and relief that may follow crying. We then provide theoretical and empirical support for our general hypothesis that crying is a self-soothing behavior by presenting and evaluating the possible physiological, cognitive, and behavioral mechanisms that may play a mediating role in the relationship between crying and homeostatic regulation that includes mood improvement and relief. Starting from the idea that social-soothing and self-soothing mechanisms share the same physiological systems, we propose that biological processes act in parallel with learning and reappraisal processes that accompany crying, which results in homeostatic regulation. Given the parallels between self-soothing behaviors in humans and animals, we also propose that crying might self-soothe through a mechanism that shares key properties with rhythmical, stereotypic behaviors. We conclude that, in addition to the importance of socially mediated mechanisms for the mood-enhancing effects of crying, there is converging evidence for the direct, self-soothing effects of crying.

## INTRODUCTION

The current contribution provides a state-of-the-art overview of the scientific literature regarding self-soothing effects of crying and presents specific hypotheses concerning the determinants of these effects and the putative involved mechanisms. We start with the definition of crying and description of its intra- and inter-individual functions, which is followed by a brief reflection on the concept of self-soothing, how it can be defined, and how it relates to several kindred concepts. Here we also underline the distinction between social-soothing, which refers to the soothing effects of the comfort and social support provided by others, and self-soothing, which refers to the *direct *effects of crying on homeostatic processes of mood regulation and stress reduction of the crier. We continue by describing why research has yielded inconsistent findings with respect to the effects of crying on mood and the related homeostatic processes in the crying individual. We try to explain the possible sources of these variations, ranging from crier characteristics, crying antecedents, the manifestations of crying, and reactions of others, to important methodological issues. Subsequently, in the central part of our paper, the focus is on our main hypothesis that crying also directly results in mood enhancement and promotes return to homeostasis. Mood changes following crying have typically been operationalized (although often rather vague and not explicitly) as a return of mood to baseline (or even above) after an initial deterioration that accompanies or precedes the onset of crying. Our approach is conceptually limited to mood returns to baseline, that is, homeostatic processes (but not necessarily without further enhancements that end up above the baseline). Such conceptualization thus also includes a return from positive mood to baseline values. We discuss physiological, cognitive, and behavioral mechanisms that might underlie such self-soothing effects of crying. Along these lines, we present more specific hypotheses about the mechanisms through which self-soothing effects of crying are mediated, which imply increases in the activation of parasympathetic nervous system (PNS) and oxytocin (OT) levels that are coupled with cognitive (e.g., reappraisal) and behavioral (e.g., sobbing) processes.

## HUMAN CRYING AND ITS FUNCTIONS

Crying has been defined as a complex secretomotor phenomenon characterized by the shedding of tears from the lacrimal apparatus, without any irritation of the ocular structures, and often accompanied by alterations in the muscles of facial expression, vocalizations, and in some cases, *sobbing*, which is the convulsive inhaling and exhaling of air with spasms of the respiratory and truncal muscle groups (Patel, 1993). This universal and uniquely human emotional expression can be elicited by a plethora of events, from those seemingly mundane and unimportant to the most crucial events in one’s life, and ranging from extremely negative to extremely positive experiences (Vingerhoets, 2013). For example, watching a movie or enjoying the beauty of nature may both make an individual tearful, just as the passing away of attachment figures or birth of a child. Crying occurs predominantly in situations characterized by separation, loss and helplessness, and being overwhelmed by strong emotion, be it negative or positive (Vingerhoets, 2013).

Crying serves two broad categories of functions. *Intra-individual* functions of crying (e.g., Breuer and Freud, 1895/1955; Frey, 1985) refer to the effects that crying has for the crying individual him/herself. These intra-individual functions are predominantly linked to stress reduction and the experience of mood enhancement and relief that follows crying, making them important for the concept of self-soothing (see below).

*Inter-individual *functions, in contrast, concern the effects that crying has on other people. Theories that emphasize these social effects of crying (e.g., attachment theory; Bowlby, 1980; behavioral evolution theory; Hasson, 2009) stress the signal value of distress vocalizations and/or of human tears. From the perspective of attachment theory, crying is viewed as an appeal for the presence and attention of the caregiver (Nelson, 2008). More recent hypotheses on the functions of human crying (see Walter, 2006; Hasson, 2009; Trimble, 2012; Vingerhoets, 2013) emphasize that crying (and especially visible tears, because only their effects have been investigated) promotes empathy and prosocial behavior, including stimulation of caregiving and protective responses from others, facilitates social bonding, and reduces inter-personal aggression. Recent research has indeed shown that visible tears have a considerable impact on the evaluation of a human face, the identified need for support, and the self-reported willingness to provide assistance and comfort of observers (Hendriks et al., 2008a; Provine et al., 2009), even at the automatic, pre-attentive level (Balsters et al., 2013). On the other hand, in particular acoustical crying (of infants) may also elicit anger, irritation, frustration, and even aggression from others (Vingerhoets, 2013). However, we currently do not yet fully understand from whom and under what conditions negative reactions to crying may be expected.

Until now, there has not been any attempt to connect each of both postulated functions to one specific component of crying (i.e., tears, vocalizations). Rather, the question was whether the crying process as a whole brings relief or whether it might influence the behavior of others (although in recent studies the focus was exclusively on the role of visible tears). Such an approach nevertheless may make sense; for example, one may wonder if the acoustical (distress vocalizations) and visual (tears) components mainly serve communicative purposes, and whether the sobbing is more important for mood and homeostatic regulatory functions. However, as we will outline in this contribution, there are certain reasons to believe that these three components may serve similar inter-individual and intra-individual functions (at least to a certain extent), although the mechanisms through which they result in self-soothing effects may just partly overlap.

To better understand the effects of crying in humans, it is also important to consider the animal counterparts to human crying (in the domain of communication), as well as other animal behaviors that might serve stress-reducing functions. In all mammals and most birds, offspring react with separation calls or distress calls to being removed from the parents. There can be little doubt that this is the phylogenetic basis of the acoustical crying of human infants. This very basic form of crying is meant to undo the separation from the parents and it is not likely to have any direct soothing function. Rather, this behavior seems to be associated with a state of increased arousal and distress, although it may ultimately result in soothing because of the comfort and support it elicited, that is, because it has fulfilled its inter-individual function. In animals, distress calls are mainly displayed by young offspring, and they are never accompanied by the production of tears. On the other hand, candidates for the mechanisms that might contribute to reduction of distress in non-human animals (and in humans as well) can be found in *displacement behaviors* and *stereotypies*, which are proposed to serve communicative functions as well (Maestripieri et al., 1992; Troisi, 2002).

## WHAT IS SELF-SOOTHING?

Intra-individual functions of crying cannot by definition be considered equivalent to self-soothing because there are different paths through which crying may affect a crying individual, or, more specifically, reduce his/her distress. Thus, we will first define the concept of self-soothing and explain its position in relation to other aspects of *emotion regulation*. We consider self-soothing to be a form of emotion regulation, which includes extrinsic and intrinsic processes involved in monitoring, evaluating, and modifying emotional reactions (Thompson, 1994; Gross, 1998). Under the term *self-soothing*, we subsume all self-directed behaviors and internal processes that are aimed to calm an individual in distress, that is, to diminish primarily negative emotion and corresponding physiological arousal, eventually resulting in homeostasis.

Gross (1998) makes a major distinction between *antecedent-focused* and *response-focused* emotion regulation. Antecedent-focused emotion regulation refers to cognitive processes and behaviors that are present before an emotion response has been initiated, such as the selection of situation, active changing of the situations, but also reappraising the meaning of a situation, etc. Response-focused emotion regulation, in contrast, refers to the process of dealing with one’s emotions after the onset of the emotion process that includes behavioral tendencies, physiological arousal, and subjective experiences. Self-soothing can thus be considered as a response-focused emotion regulation strategy, because it modulates one’s negative emotional experience and/or excessive physiological arousal linked to emotion, whether it is positive or negative. However, note that most of the processes or strategies that are subsumed under the antecedent-focused emotion regulation can also be applied when an emotional response is already present. For example, when an individual wants to find relief from a negative emotion, (s)he can try to reappraise the meaning of the situation, to change how (s)he feels.

According to the psychological stress theory (Lazarus, 1993), self-soothing may be regarded as a kind of *emotion-focused coping*, which, similar to response-focused emotion regulation, refers to behaviors and cognitions applied to deal directly with unwanted emotions. Along these lines, when people experience strong negative or even extremely positive emotions, they may engage in a variety of adaptive or maladaptive behaviors to reduce such feelings. For example, people may try to distract themselves, try to relax, engage in physical activities, talk to a friend or family member, think about something positive, use drugs or alcohol, etc. (the latter behaviors are also often used to deal with extremely positive feelings). All these activities may be subsumed under the term *mood management,* which refers to self-soothing behaviors aimed at influencing one’s mood (e.g., Thayer et al., 1994). In infants, self-soothing may also consist of self-manipulative behaviors, such as thumb sucking, fingering clothing, and twirling hair (Diener et al., 1997). A similar function is subsumed under the term *mood repair*, which specifically refers to attempts to reduce feelings of sadness or dysphoria using a variety of self-regulatory cognitive, behavioral, and inter-personal strategies (see Parkinson and Totterdell, 1999, for review).

A final relevant concept that has often been discussed in the crying literature is *catharsis. *Catharsis refers to the experience of relief resulting from the expression of strong emotions that may be experienced after, for example, crying, swearing, or aggressive acts. The general idea is that such expressions result in the release of excessive emotional energy that, if not adequately released, might convert into a variety of psychological and even physical health problems (Breuer and Freud, 1895/1955). Catharsis thus by definition implies self-soothing, because it brings relief and diminishes tension and/or negative feelings irrespective of the removal of the external source of stress. While mood management refers to intentional behaviors displayed with the aim to reduce one’s distress, catharsis is not a behavior but the postulated effect of the expression of strong emotions, often implicitly assuming a physical energy model (draining of emotional energy, safety valve model).

**FIGURE 1:**
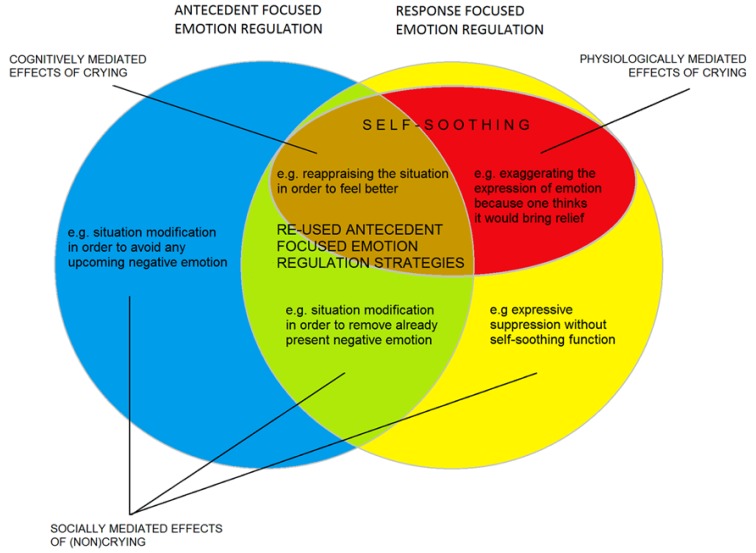
**Self-soothing in relation to antecedent and response-focused emotion regulation strategies**.

In sum, in the psychological literature there is ample attention to a variety of behaviors and cognitions aimed at modifying one’s emotional or mood state. Our focus will be on behaviors that are displayed to reduce feelings of mental or physical distress. For the sake of precise definitions, we will draw a line between emotion regulation behaviors that directly change the *situation* (and additionally may, as a consequence, change the experience) and behaviors and internal processes that directly change the *experience* (and may indirectly change the situation). We apply the term *self-soothing* only to the latter group of behaviors. Following such a definition, also reappraisal techniques, which are subsumed under antecedent-focused emotion regulation by certain theorists (e.g., Gross, 1998), can be regarded as self-soothing strategies (see **Figure 1**). On the contrary, behaviors that result in decreasing of one’s distress because they elicit help and comfort form others (*social-soothing*) do not enter this category. Thus, if it would be established that crying results in decreasing of distress only because it elicits soothing from other people, that would mean that it is not a self-soothing behavior, but rather a form of inter-personal emotion regulation.

## CRYING AND SELF-SOOTHING

The main hypothesis in this contribution is that crying serves self-soothing functions, but that this effect can be facilitated, mitigated, or completely neutralized by several moderating external factors (e.g., the experience of embarrassment or comfort induced by others). Relatedly, one may further wonder which mechanisms are responsible for these effects, or which of the specific components of crying itself (production of tears, distress vocalizations, or sobbing) or of the crying context ultimately produce the self-soothing effects. Self-soothing effects of crying imply the returning of both subjective, emotional states (mood) and physiological arousal to baseline (i.e., pre-crying or better pre-crying inducing stimulation) levels. Thus, we expect self-soothing effects of crying to be the consequences of homeostatic regulation that are promoted by crying.

### CRYING AS A MOOD-ENHANCING BEHAVIOR

While the limited scientific literature on the functions of crying is more convincing with respect to the inter-individual than to the intra-individual effects of crying, remarkably, in folklore and the popular media the notion that crying particularly serves intra-individual effects is much more prominent. The idea of cathartic effects of crying is rather old, dating back to classical antiquity (Vingerhoets, 2013). For example, the Roman poet Ovid already wrote that “It is some relief to weep; grief is satisfied and carried off by tears.” And a review of popular American and British magazines from 1848 until 1985 revealed that people undoubtedly believed that crying is good for one’s mental and physical health (Cornelius, 1986). Darwin (1872/1965) also pointed out that children may cry to experience relief resulting from crying as from “any great exertion” (p. 174). He even suggested a dose–response relationship between crying and relief, meaning that more intensive crying would result in greater relief. There is thus a great general willingness to attribute self-soothing qualities to crying.

Claims about the beneficial effects of crying on health and subjective feelings can also be found in the current popular psychology literature (for review, see Hendriks et al., 2008b). The lay acceptance of the idea that crying serves self-soothing purposes is further demonstrated in a recent study by Simons et al. (2013). When asked about the reasons why they sometimes deliberately enhance their own crying in sad or upsetting situations, the respondents reported that this was mostly driven by intra-personal motives. In other words, when people deliberately stimulate their own crying, for example, by focusing on particular memories or by modulating facial expression, they report to do so often because of the anticipated effects on themselves, rather than because of the possible effects on people around them (however, see also later on). Crucially, the most often reported reasons to up-regulate crying were centered on decreasing one’s distress. While it cannot be ruled out that participants were reluctant to admit more Machiavellian motivations to up-regulate their crying, this finding nevertheless suggests not just wide acceptance, but also a lay implementation of the idea of self-soothing functions of crying in everyday life.

More modern thinking about the intra-individual functions of crying started with psychodynamic theories, which claimed that the expression of emotion in general brings relief (catharsis), whereas, on the other hand, the inhibition of expression may promote the development of various kinds of (psycho)somatic and mental health problems (e.g., Breuer and Freud, 1895/1955). Subsequent theoretical approaches to crying relied heavily on these psychodynamic premises and regarded the absence of crying when it would be expected as defensive (e.g., Wallerstein, 1967) or symptomatic (Lindahl, 1977). For a long time, the most frequently applied psychotherapeutic technique consisted of the interpretation of defenses and active encouragement of crying (Nelson, 2008). Currently, a significant majority of psychotherapists still actively encourage their clients to cry (e.g., Trezza et al., 1988; Nelson, 2008).

### INTRA-INDIVIDUAL EFFECTS OF CRYING: EMPIRICAL FINDINGS

Despite its pervasiveness, the popular claim that crying has intra-individual, and specifically, self-soothing effects, cannot be uncritically accepted when considering the current state of the relevant scientific literature. The empirical record with respect to the question of whether or not crying brings relief and improves mood reveals highly discrepant findings. Discrepancies in these findings are seemingly largely dependent on the applied research methodology.

In retrospective studies, participants are typically asked to remember and report how they actually felt after their most recent crying episode. In one such study that seems to leave little doubt about the benefits of crying for mood, Bylsma et al. (2008) found that most of the men and women in 35 countries reported feeling better after crying, whereas in a everyday, diary study (Bylsma et al., 2011), the percentage of crying episodes that reportedly was associated with beneficial effects was approximately 30%.

In contrast, quasi-experimental laboratory studies, in which crying was induced by exposing participants to sad films, have consistently demonstrated greater decreases in mood immediately after the film in participants who cried compared to those that did not cry but watched the same film (e.g., Kraemer and Hastrup, 1988; Martin and Labott, 1991; Gross et al., 1994; Rottenberg et al., 2002; see Cornelius, 1997 for a review). Moreover, these negative emotional consequences are preceded by increased activation of the sympathetic nervous system, which is typical for distress (Rottenberg et al., 2002). Also, in retrospective and diary studies, self-reported mood change following crying episodes varies considerably among individuals (Lombardo et al., 1983; Bylsma et al., 2008, 2011). This variability in post-crying mood is nicely illustrated by the results of the Simons et al. (2013) study. In the domain of intra-individual motives to regulate crying, study participants surprisingly reported basically the same motives for both enhancing and preventing their own crying: to avoid or decrease distress. In other words, some individuals apparently expect crying to increase their distress, whereas others expect the opposite.

**FIGURE 2:**
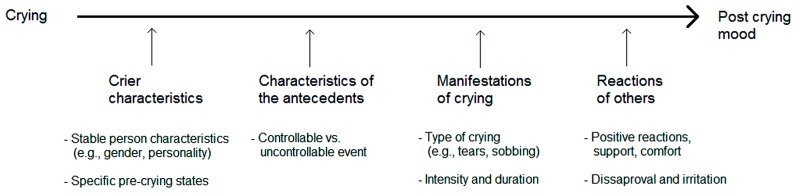
**Moderators of the effects of crying on post-crying mood**.

Given such a strong variation, Rottenberg et al. (2008a) came to the conclusion that the better formulation of the question “Does crying bring relief?” would be: “Under what conditions and for whom is crying likely to be beneficial?” These authors subsequently analyzed the possible reasons for the observed variability in the mood effects of crying and concluded that the variations in the results depended on the following factors, which are summarized in **Figure 2**: (1) crier characteristics, (2) characteristics of the antecedents, (3) manifestations of crying, and (4) reactions of others. It goes without saying that for a good understanding of the correlates of individual differences in mood improvement following crying, we need to know more about the involved mechanisms. On the other hand, the understanding of the individual differences in self-soothing effects of crying may help to better understand the workings of the mechanisms through this may happen, which is the central topic of this contribution. Finally, to understand the mechanisms through which crying may self-soothe, it is also important to consider the cases in which crying may result in stress reduction, return to homeostasis, and mood improvements in which it still cannot be considered as self-soothing. The following section is directed to such sources of variation, as well as to factors that may facilitate, mitigate, or completely neutralize self-soothing effects of crying. In addition to the sources of variation (depicted in **Figure 2**), we will here also consider the methodological issues that may account for the observed variability.

#### Crier characteristics

***Stable person characteristics.*** Stable person features include both biological (e.g., gender) and psychological characteristics (e.g., personality traits, clinical characteristics). Changes in mood that follow a crying episode are found to be dependent on both kinds of stable characteristics. What do we currently know? A large international study revealed that men report slightly smaller mood improvements after crying than women (Becht and Vingerhoets, 2002). However, this effect was not found in other studies with fewer participants (e.g., Peter et al., 2001).

With regard to personality features, of the major (i.e., Big Five) personality dimensions, only extraversion proved to be positively correlated with self-reported relief and positive feelings after crying, while certain aspects of conscientiousness seem to have the opposite effects (De Fruyt, 1997). Interestingly, those who reportedly experienced more negative effects of crying also had higher scores on the conscientiousness facet dutifulness. The author interpreted these findings as perhaps the consequence of guilt and shame that may follow one’s crying, as this trait is related to adhering to strict behavioral rules, at least in certain cultural contexts, such as those that prescribe the suppression of crying in public settings.

In the clinical domain, depressive and anxiety symptoms, anhedonia, and alexithymia were all found to be related to worsened post-crying mood (De Fruyt, 1997; Rottenberg et al., 2008a), with the latter factor being the strongest predictor of mood deterioration. Perhaps, alexithymics’ lack of insight into the causes and meanings of their crying may perpetuate their negative mood after crying. There can be little doubt that the effects of personality, at least partially, also are mediated by reactions of others (social-soothing) as well as by physiological, cognitive, and behavioral (self-soothing) mechanisms that we will present below. For example, the observation that extraverts report more mood improvement may result from the fact that extraverts have more social skills and are better able to elicit social support, that is, to benefit from the inter-individual functions of crying. On the contrary, the fact that men report less mood improvement than women may be connected with the fact that men also report feeling more embarrassed and ashamed than women when crying (Van Hemert et al., 2011; Vingerhoets, 2013). In this case, the otherwise existing self-soothing effects of crying may be precluded through socially created obstacles. Finally, the cases of alexithymics and depressive individuals stress the importance of (malfunctions in) cognitive and physiological mechanisms that may be responsible for self-soothing (see below).

***Specific pre-crying states.*** Given the co-occurrence of both worsened post-crying mood and the inability to cry in some depressed individuals (Rottenberg et al., 2008b), one could argue that the very same mental/emotional states that make an individual capable of crying are also important determinants of subsequent mood changes. The possibility that the transitory capability to cry rather than crying itself is a determinant of post-crying mood improvement also fits well with the attachment-based approaches in psychotherapy. According to Nelson (2008), an adult with a secure style of attachment is able to activate crying, an attachment behavior, when vulnerable, because (s)he is capable of intimacy and comfortable with the soothing behavior it evokes in others. In a similar vein, the beneficial effects of crying on one’s mood may in fact represent the satisfaction of achieving the state in which an individual is able to activate the attachment behavior. Recent theoretical and empirical work has shown that attachment states may actually vary considerably over time, or, to put it differently, attachment styles in an individual are constantly changing (e.g., Xu and Shrout, 2013). It could thus be hypothesized that the self-soothing effects of crying occur only when an individual is in a state of secure attachment.

#### Characteristics of crying antecedents

Given the frequency of their occurrence, people seem to cry much more often for rather mundane and everyday situations (arguments, minor failures, reproaches) than because of severe emotional events (death, divorce, romantic break up, victimization, etc.), which generally occur with a very low frequency. In adults, reading poems and watching documentary reports and movies as well as listening to music are also among the major reasons for crying. In addition, especially for females, conflict situations are important (Vingerhoets, 2013). Here, the controllability of a situation appears to be an important predictor of the mood following crying. For example, mood improvements were found to be more often experienced if a crying individual reported that s/he him/herself was responsible for the crying episode. In contrast, if strangers or family/relatives were responsible for the crying episode, this was negatively related to mood improvements (Bylsma et al., 2008). Witnessing suffering of other people as a reason for crying was also found to be negatively related to mood improvement (Bylsma et al., 2008), which may also be explained by the reduced experience of control in such situations. Research has also shown that people more often experience mood improvements after crying when, as one would expect, events that elicited their crying were resolved (Cornelius, 1997; Bylsma et al., 2008). However, while these findings may explain when crying might be followed by mood enhancements, they do not answer the question whether and how crying may directly reduce distress.

#### Manifestations of crying

Crying may vary in duration, intensity, and in several qualitative aspects. For example, it may vary in both the involvement and intensity of specific features (sobbing, distress vocalizations, tearing). As already said, perhaps different characteristics and components of crying may represent different mechanisms that mediate the relationship between crying and mood. For example, one could ask whether sobbing has direct, self-soothing consequences, whereas tears only result in mood improvements through the positive reactions of other people. However, while there might be certain differences in the reactions of others depending on the different components (or types) of crying, these different components may, as we claim, also directly improve one’s mood through the similar physiological and cognitive mechanisms as part of the same process (see below). Also, the benefits of crying may be precluded if its loudness or duration is perceived as annoying and consequently elicits negative responses from the others. Furthermore, sobbing that exceeds some moderate time period may require a considerable amount of energy expenditure that finally results in tiredness (and eventually sleep) or in worsened mood.

Until now, research on the effects of the specific type of crying on subsequent mood change is non-existent, while there is only one study addressing the relationship between the intensity and duration of crying with subsequent reported mood improvement. Bylsma et al. (2011) demonstrated that more intense crying was associated with greater post-crying mood improvement, whereas duration of reported crying episode appeared to be irrelevant. Future studies, which specifically address this issue, are needed to unravel the possible role (and mechanisms) of different components of crying. In particular, the distinction between protest crying and sad crying, as made in the attachment theory (e.g., Nelson, 2008) deserves special attention, because each of them has a different character. In addition, new studies would benefit from designing new, technologically more advanced, objective measures of crying intensity, which are currently badly lacking.

#### Reactions of others

**FIGURE 3:**
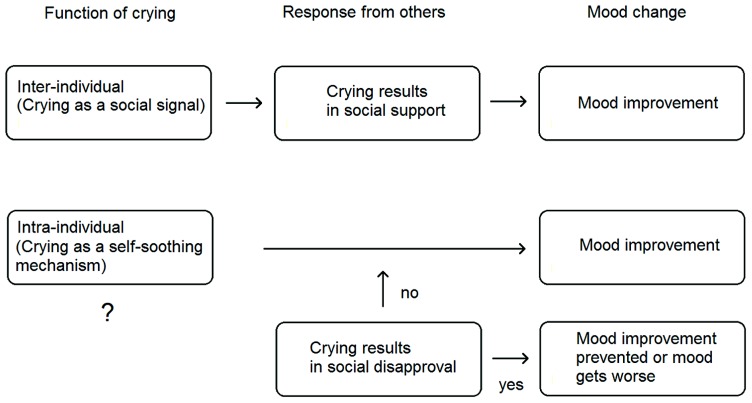
**A model of socially mediated and moderated effects of crying on mood**.

Given the strong inter-individual effects of crying, one should not overlook their possible major role in the beneficial mood effects of crying. The mental and physical well-being of the crier may be improved primarily through the elicited comfort and succor (Vingerhoets et al., 2000, 2009; Hendriks et al., 2008a; Nelson, 2008). Benefits of crying may also be realized by reducing aggression in others and facilitating inter-personal conflict resolution (see Vingerhoets, 2013). Inter-individual effects of crying may thus mediate, but they may also moderate the relationship between crying and mood. That is, they may result in mood improvements by fulfilling certain inter-individual functions of crying as stated above, or, on the contrary, they may prevent the otherwise automatically elicited mood improvements after crying (self-soothing) if there are inter-individual obstacles (e.g., crying resulting in embarrassment or shame; Figure 3).

Partial support for the mediating role of inter-individual effects of crying for the subsequent mood was found in studies which showed that criers who received social support while crying were more likely to report mood benefits than the criers without support (Cornelius, 1997; Bylsma et al., 2008). Unfortunately, such findings do not fully discriminate between the possible mediating and moderating role of inter-individual effects. In addition, we do not know the details of what the social support actually included. Was it more physical comforting? Empathic soothing words? Or providing information and advice? Or did the criers receive practical help to solve their problem? What we do know is that when asked about the inter-individual motivations to up-regulate crying, people often report that they enhance their crying because they want others to know how they feel, because they need support from other people, and because they feel that others’ reactions will decrease their distress (Simons et al., 2013). Thus, there is some empirical support for the hypothesis that crying may improve mood through other people’s positive responses (providing succor and comfort).

On the other hand, self-soothing effects of crying might be precluded if the crier deems his/her crying inappropriate in a given social context or in general. The experience of negative social emotions like shame and embarrassment (e.g., due to the presence of some specific others) may prevent post-crying mood benefits (Becht and Vingerhoets, 2002; Bylsma et al., 2008). The felt appropriateness of one’s crying undoubtedly depends on various individual difference variables as well as on the reactions of others, which may be determined, among others, by cultural factors (Becht and Vingerhoets, 2002). For example, negative feelings regarding one’s own crying may be the consequence of social anxiety or the explicit disapproval by others (see Cornelius, 1997, 2001 for an overview). Such findings are also compatible with those of the Simons et al. (2013) study, where in 33% of all reported down-regulating cases of crying, participants reported both “not wanting to cause distress in others” and “not wanting to increase one’s own negative feelings,” which suggests that mood deterioration after a crying episode might result from having caused distress in other people. This especially can be observed in parents when trying to prevent their children witnessing their crying and distress.

Along these lines, Bylsma et al. (2011) found that the presence of one single person (in most cases, this is an intimate, such as one’s mother, friend or romantic partner) during the crying episode was most likely followed by the improvements in mood of the crier, whereas the presence of more than one person was associated with negative mood effects. The authors speculated that the presence of a larger number of other individuals might increase the likelihood that crying induces shame and embarrassment, while the presence of just one intimate more likely may result in mood improvements via comforting and consolatory behaviors.

To summarize, in addition to the obstacles that the negative reactions of others may pose for the putative self-soothing effects of crying, there is certain evidence that homeostatic and mood-enhancing effects of crying may be the consequence of social-soothing. This is further important because, central to our claims, there is a possibility that putative self-soothing effects of crying may be based on the neural/physiological mechanisms that are shared with those involved in social-soothing.

### DISCREPANCIES BETWEEN RETROSPECTIVE AND QUASI-EXPERIMENTAL STUDIES

The sources of variation that are related to social environment and crying antecedents may also partly explain the apparent discrepancies in the observed effects of crying on mood in retrospective and laboratory studies. For example, the specific laboratory setting is typically devoid of various features that might be crucial for the soothing effects of crying, such as social support and comforting (Cornelius, 2001; Rottenberg et al., 2008a), and is characterized by artificial conditions, in which participants are video recorded or observed by strangers, which may induce embarrassment in criers (Rottenberg et al., 2008a). Furthermore, the exposure to emotional films, the standard laboratory method for the elicitation of crying, is characterized by a lack of control, because the participants’ behavior, including their crying, does not have any influence on the outcome of the depicted situation. This is in contrast to real life, where crying may often have a considerable impact on the situation.

Furthermore, it is arguable whether in these two types of studies (i.e., quasi-experimental vs. retrospective), exactly the same concept is measured. When retrospectively reporting mood changes that followed their crying, people likely are biased by implicit lay theories, which pose that crying brings mood improvement and relief (Cornelius, 1997). Relatedly, it cannot be ruled out that people more easily remember crying episodes that are associated with mood improvement or that they are more willing to report positive than negative crying experiences in retrospective studies (Cornelius, 1997).

A further very simple explanation for the found inconsistencies stresses the role of memory biases. Since mood in criers, compared to non-criers, reaches a nadir after a crying episode, the necessary consequence of this change is the return to baseline that must happen sooner or later. This recovery process can also be experienced as very intensive and, as such, it may be misperceived as a real mood improvement. People thus may have the experience of feeling better after crying, because compared to non-crying situations they indeed experience a major mood change, although this may not represent a real improvement (in comparison to a situation without crying. Closely related to this, in practically all quasi-experimental studies mood was assessed immediately after the crying induction (e.g., exposure to a sad film), which raises the question whether the beneficial intra-individual effects of crying perhaps need some more time to develop (Rottenberg et al., 2008c; Vingerhoets, 2013). Since the exact timing of the beneficial effects of crying that are reported in retrospective studies is impossible to reconstruct accurately (Rottenberg et al., 2008a), the most appropriate research strategy in future laboratory studies is to induce crying and to take multiple mood measurements over an extended period of time following (non)crying episodes. Finally, mood is often measured with self-reports that not always accurately reflect the internal states of the subjects (e.g., see Nisbett and Wilson, 1977). Thus, future experimental research should employ behavioral and physiological indicators of mood as well (e.g., posture or voice modulation and variations in cardiovascular activation, see below).

In conclusion, we cannot uncritically accept the seeming beneficial effects of crying on one’s mood as a valid support for our hypothesis about self-soothing effects of this behavior. To adequately evaluate the hypothesis that crying has self-soothing properties, all these factors should be taken into account as possible moderators, since their impact hardly fits our definition of self-soothing. Nevertheless, there are still some good reasons to seriously consider the hypothesis that (specific components of) crying may induce actual mood improvement and to elaborate on possible underlying mechanisms. The consistent findings of quasi-experimental studies, which all show mood deterioration after crying, strongly suggest that the possible positive effects need some time (at least several minutes) to develop. Which mechanism(s) may be responsible for how the initial decrease in mood develops into a possible mood improvement?

### SELF-SOOTHING MECHANISMS OF CRYING

If crying is to be considered a real self-soothing behavior, then it should also result in mood improvements when crying alone, with no others present. We further propose that these mood returns to baseline may comprise the activation of different neural systems such as social engagement system (Porges, 2003a, b) and variations in oxytocin (OT) levels, that have an important role in social-soothing as well (see later). Thus, we will direct our attention to the role of the *physiological*, *cognitive,* and *behavioral* mechanisms that may be responsible for such effects (See **Table 1**). Note, however, that each category of mechanisms can be supported or accompanied by workings of the mechanisms from the other categories, which makes things more complex. For example, physiological processes promoting soothing might in fact represent the consequence of cognitive or behavioral changes that accompany crying. This may typically happen during reconciliation, where both the reappraisal of the social context as less threatening (or more accepting) than before, and a burst of sobbing might result in increases in PNS activity and OT (and possibly opioid) levels,followed by mood enhancements. Again, note that such effects do not necessarily require any further response from social environment (while they may clearly evoke them).

**Table 1:** Proposed mechanisms that underlie the direct relationship between crying and mood improvement.

Category	Mechanism
*Physiological*	Parasympathetic nervous system activity
	Blood clearance and detoxification
	Opioid release
	Nerve-growth factor release
	Oxytocin
	Changes in central nervous system activity
	(e.g., neurotransmitters, blood flow)

*Cognitive*	Awareness of one’s own tears
	Self-image improvement
	Achieving a new perspective on or resolution to a sad event
	The role of learning

*Behavioral*	Sobbing as a rhythmical behavior with stereotypic properties

The effects of crying might also generally correspond to recently described phenomenon: emotional numbness observed after social rejection and psychotrauma. According to the “numbness hypothesis” (Twenge et al., 2003), individuals who are being ignored or excluded or those who have experienced a traumatic experience (Litz, 1992) may become emotionally numb and may not show any overt signs of distress. These phenomena illustrate that humans seem to have the capacity to react to the exposure to (extreme) stressors with a state that prevents the occurrence of strong negative emotions. The question is whether crying may be helpful to reach such a state as well. Such processes may at first seem to differ from those directly involved in return to homeostasis, but it has to be said that such distinction is unnecessary, because, as we will present below, self-soothing mechanisms of crying might operate by both decreasing existing stressful response as well as by preventing incoming stress response, both finally resulting in homeostasis.

#### Physiological mechanisms

The moment at which an individual’s state shifts from more active engagement to powerless signaling for help when crying may be accompanied by shifts in various autonomic, neuromuscular, and neurobiochemical systems that as a whole may indeed induce relief. But which specific mechanisms are involved? Below we briefly discuss theoretical and empirical arguments for the involvement of some specific mechanisms.

***Parasympathetic nervous system activity.*** Activation of PNS accompanies states of rest and restoration of an organism. It is also related to social engagement (Porges, 2003a, b) and is expected to be regulated according to *social baseline* levels, which also implies cardiovascular homeostasis in response to adequate levels of (perceived) social support (Beckes and Coan, 2011), or social-soothing. Early studies on the relationships between human crying and changes in PNS activation focused on the distress cries of newborns, demonstrating that crying was related to *de*creases in PNS activity (Porter et al., 1988). However, in these studies PNS deactivation was most probably the consequence of a stressful event and seems to have resulted in, rather than resulted from crying. Furthermore, distress vocalizations of newborns and (tearful) crying of adults should not be simply considered equal (see also Nelson, 2008). It is thus hard to compare these findings with those indicating that tearful crying in adults is accompanied with and possibly is followed by *in*creases in PNS activation (e.g., Rottenberg et al., 2003; Hendriks et al., 2007). Crucially, variations in PNS activity associated with crying may vary depending on when in the time course of a crying response they are measured. Hendriks et al. (2007) observed both physiologically arousing and calming effects of crying, with the calming effects (e.g., slowed breathing) lasting 2–3 minutes longer than arousing effects (e.g., increased heart rate). Since, as already noted, there is support for the notion that elevated PNS activation is related to states of relaxation and return to homeostasis after the experience of intensive negative emotion (Porges, 2003a, b), it seems likely that increases in PNS activation observed in adults may represent a mechanism that mediates the relationship between crying and mood improvement.

Research on the relationship between physiological and emotional abnormalities in depression represents an important source of information that may enhance our understanding of the relationship between crying and subsequent mood changes. For example, Rottenberg et al. (2003) found increases in PNS activity immediately after the exposure to a tear-eliciting film in non-depressive, but not in depressive, criers, which is in accordance with the larger literature on PNS abnormalities and diminished prefrontal reactivity in depressed individuals (Rottenberg et al., 2007; Schiller et al., 2013; Bylsma et al., 2014). Although Rottenberg et al. (2003) did not examine mood changes following crying, other studies have, as already mentioned, indicated that depressed individuals tend to experience less mood improvement following crying (e.g., Rottenberg et al., 2008b), and are generally characterized by blunted emotional reactivity (Bylsma et al., 2008). Together, this suggests that diminished PNS reactivity in these individuals could account for the absence of normative PNS-mediated self-soothing effects of crying. If future studies confirm the importance of the PNS activation for mood improvement following crying by experimentally manipulating reappraisal and provided support, the next step would be to examine whether these changes are a direct consequence of (one specific component of) crying and whether they accompany some specific cognitive or social processes.

Unfortunately, it is not easy to determine whether crying stimulates the activity of the PNS, or, the other way around, whether increased PNS activation promotes crying, because it is difficult to establish the precise onset of crying. Nevertheless, there is sufficient theoretical and empirical ground to hypothesize that the self-soothing effects of crying are due to changes in PNS activity. Furthermore, since prior psychophysiological studies with adults explicitly used the elicitation of tears as the criterion whether a participant had cried (rather than on sobbing or distress vocalizations), current data cannot yield the definitive answer whether the PNS mediation is connected specifically and uniquely with tearful crying. Thus, we propose that the other components of crying produce similar changes in PNS activity (see the accounts on sobbing, below).

Accumulating evidence suggests that PNS activation is associated with changes in specific prefrontal brain areas, the activity of which is inversely related to amygdala activity, or, on a more global basis, to the activity of limbic structures. Support for this model has been found in neuroimaging, neurochemical, and lesion studies (for reviews, see Davidson, 2002; Porges, 2003a; Lane et al., 2009), which also fits the notion that PNS activation may mediate self-soothing effects of crying, due to the (de)activation of the same inhibitory prefrontal structures and limbic structures that underlie threat responses. Furthermore, the cortical control of the PNS activation is based on *social engagement system *(Porges, 2003a)*, *which consists of phylogenetically newer brain structures specific to mammalian species. This system is characterized by both social (inter-individual) and metabolic (intra-individual) functions, allowing mammals to (temporarily) dampen the fight, flight, and freeze responses, and rather to engage in pro-social behavior. We postulate that in humans such inhibitory functions were *exapted* and put to a new use (for a discussion on *neural reuse* see Anderson, 2010) in situations in which an individual signals a need for help and succor, that is, when crying. These neural functions are activated when an individual anticipates (e.g., because of learned responses or self-image improvements; see below) social support and/or acceptance (i.e., social-soothing).

***Blood clearance and detoxification.*** The hypothesis that crying brings relief and even improves health because it clears the blood of toxins and pollution dates back to classical times (Murube, 2009). The biochemical variant of this claim, which has been introduced in the 1980’s (Frey, 1985), posits that crying influences well-being through the elimination, via tears, of stress hormones (e.g., cortisol) and toxic substances from the blood. In a test of this hypothesis, Vingerhoets and Kirschbaum (1997) measured the levels of salivary cortisol in women before and after watching an emotional movie. The levels of this stress hormone indeed decreased more in women who reported more intensive crying. While this finding seems to support Frey’s (1985) notion of clearance and detoxification of the blood via the tears, the fact that similar effects were observed after separation from the mother in young rhesus monkeys that do not produce emotional tears, but merely emit distress calls, seriously challenges the biochemical detoxification role of tears (Bayart et al., 1990). Such a finding thus indicates that decreases in cortisol levels are not necessarily the consequence of removal of this hormone through tearing. However, both findings suggest that the expression of distress, independent of its specific mode, may result in physiological changes that reflect decreases in stress reaction and support the hypothesis that crying may have direct self-soothing effects, although not likely through the removal of waste products and stress hormones via tears, but rather through other physiological and/or cognitive or behavioral mechanisms that are related to inter-individual (i.e., signaling) functions.

***Opioid release.*** Endogenous opioids are well known for their beneficial effects in case of emotional and/or physical pain. Wubben and Vingerhoets (2008) offered an intriguing model in which the beneficial effects of crying on one’s emotional state would be mediated via the release of opioids or OT. More precisely, according to this model crying results in heightening the (physical and emotional) pain tolerance limit, creating the emotional numbness discussed above. While any self-soothing behavior may be viewed as functional, because it allows an individual to use his/her resources in a more appropriate way (e.g., by not wasting energy for excessive stress reactions), this model specifically predicts increases in (emotional) pain tolerance after having cried. Interestingly, opioids are responsible for the self-soothing effects of stereotypies in the other mammals. As we will suggest later, perhaps similar mechanisms might mediate the calming effects of sobbing in humans. However, it is less likely that OT might be involved in promoting the states of emotional numbness, since this substance might in fact sharpen social perception by promoting familiarization to novel social contexts (see the next section). Thus, a remaining intriguing question is whether crying, and especially sobbing, also induces a comparable, but less intense state of numbness mediated by opioid level changes, which may help people to endure physical and emotional pain. For example, if increases in pain threshold following a crying episode would be experimentally confirmed, it would certainly support the general hypothesis about crying as a self-soothing behavior as well as the more specific hypothesis about opioids. However, the latter one would require further investigation in realm of other, primarily hormonal but also behavioral measures.

***Oxytocin.*** In non-human primates, OT reduces the aversive quality of social stimuli (Parr et al., 2013) and suppresses the vigilance toward potential social threats (Ebitz et al., 2013). In humans, OT is found to decrease amygdala activity in response to threatening social stimuli (Labuschagne et al., 2010), to increase calmness and general sense of well-being, and to decrease anxiety and cortisol levels during socially stressful events (e.g., Heinrichs et al., 2003; Ishak et al., 2011). What is even more important for crying, OT in humans has been found to increase the experience of attachment security (Buchheim et al., 2009). Note that all these properties also correspond to both *social engagement system* functions and the regulation of social baseline discussed above.

Recent research has indicated that this hormone may signal social stress as well, thus leading to the conclusion that its putative self-soothing effects may depend on more complex interactions (Taylor et al., 2006; Tabak et al., 2011). Nevertheless, it seems plausible that higher OT levels in individuals under stress in fact reflect a kind of coping response to that stress (note the parallel with this proposed functions of crying). Indeed, OT is proposed to facilitate habituation to stress and to enable familiarization-habituation responses to stress that is a consequence of social novelty (Tops et al., 2013).

Importantly, increases in OT are theorized to directly accompany crying (Wubben and Vingerhoets, 2008) or at least to result from comforting responses that are elicited by this behavior (Vingerhoets, 2013). Indeed, previous research pointed to increases in peripheral OT following the reception of social support (e.g., Grewen et al., 2005), which may also represent a kind of normative response when inter-individual function of crying is fulfilled. Following our general hypothesis about the common mechanisms of social-soothing and self-soothing, we further expect that a similar mechanism lies at the basis of self-soothing effects of crying.

Interestingly, OT is also closely involved in the regulation of PNS activity (Snowdon and Ziegler, 2004). In this regard, it might be an interesting hypothesis that the PNS activation in its turn triggers the release of OT, with its well-known stress-relieving effects. Furthermore, since there is also some evidence that sadness is associated with low OT levels (Turner et al., 1999), increases in the level of this hormone following crying could potentially lead to subsequent mood enhancement. Thus, our claim about the mediatory role of OT goes hand in hand with the claim about the importance of PNS activation. While the precise mechanisms still need to be clarified, the findings and a general logic regarding both PNS and OT functions provide considerable support for the more general hypothesis about the existence of direct, self-soothing effects of crying. Future studies could benefit from taking into account the findings about inter-individual variations in the sensitivity to OT administration. There is some evidence that certain individual traits (e.g., self-reported parental love-withdrawal) may mitigate the beneficial effects of this hormone (for a review, see Bakermans-Kranenburg and van Ijzendoorn, 2013), further suggesting that OT-mediated effects of crying may vary along the continuum of certain individual difference variables. Thus, the hypothesis about the mediatory role of OT for the mood-enhancing effects of crying would be supported by the findings about an association between such specific individual difference variables and the degree of mood enhancements following a crying episode.

***Nerve-growth factor release.*** Nerve-growth factor (NGF), a protein proven to have an important function in the restoration of neural cells is found to be present in the lacrimal gland (Nguyen et al., 1997) as well as in tears (Park et al., 2008). Interestingly, decreased NGF levels have been found in depressed individuals (Duman and Monteggia, 2006), and there is limited evidence of antidepressant effects of NGF in non-human mammals (Altar, 1999). Given these findings, Provine (2012) hypothesizes that the mood-enhancing effects of tears can be attributed to this substance. More specifically, this author postulates that the NGF in emotional tears, which drain from the lacrimal glands through the nasal cavity back into the body, can bypass the blood–brain barrier and easily access the brain via the olfactory and trigeminal nerves (Benedict et al., 2011). This intriguing hypothesis, which is currently awaiting empirical testing, does not necessarily contradict the proposed roles of PNS and OT (and possibly opioids), and it is also in accordance with the more general hypothesis about the homeostatic, self-soothing effects of crying.

***Changes in cerebral blood supply.*** According to the vascular theory of emotional efference (Zajonc et al., 1989), specific facial muscle activity associated with emotional expressions helps to compensate for the changes in cerebral blood supply that are created by negative emotional states. This mechanism is hypothesized to maintain stability in the cerebral blood circulation, which is important for cerebral thermoregulation and mood. In addition to the various consequences of facial muscles’ activity, nasal inhalation of cool air (resulting in the cooling of the brain) is experienced as pleasurable, whereas inhalation of warm air is experienced as aversive. Since even subtle increases in cerebral temperature may have impact on the activity of emotion-linked neurotransmitters (Van Boxtel, 1997), this subsequently may result in a lowered mood. Sobbing, which often accompanies crying, is characterized by fast and successive inhalations of air that is, as a rule, colder than body temperature. Thus, according to this hypothesis, sobbing – rather than emotional tearing – might be held responsible for the improvement in mood.

For the sake of completeness, it has to be added that there is also an opposing theory, which suggests that negative facial expressions facilitate the experience of negative mood, rather than dampening it (Darwin, 1872/1965; James, 1884). While this possibility has recently received some support (e.g., Strack et al., 1988; Hennenlotter et al., 2008; however, see also Prkachin, 2005), it is not incompatible with the very specific hypothesis based on the nasal inhalation, which may have effects on brain functioning through different pathways than those resulting from the facial muscles’ activity. Again, this possibility is not in contrast with the hypotheses about the central role of PNS and OT (and opioids as well) in self-soothing effects of crying.

#### Cognitive mediation

Crying may also produce beneficial effects on mood via cognitive mechanisms that are functionally interconnected with physiological mechanisms discussed above. These may include, more specifically, awareness of one’s tears, self-image improvements, reaching a new understanding, or learning processes. All the cognitive mechanisms that we consider may be coupled with all three forms of crying, that is, tearful and vocal manifestations, as well as sobbing.

***Awareness of one’s tears.*** Is there any support for the hypothesis that the awareness of flowing tears has an effect on how we feel? In a Japanese study, researchers simulated flowing tears by dropping some lukewarm water on the cheeks near the lacrimal ducts of both eyes of their study participants (Mori and Mori, 2007). In the control condition, the same procedure was applied, but the “artificial tears” were now dropped on the temples. Subsequently, it was determined whether this procedure had any effect on mood. In support of the hypothesis, it appeared that more of the participants in the simulated-tear condition, compared to the control condition, reported sadness, leading to the conclusion that the perception of “tears” on the appropriate area of one’s face may indeed induce or strengthen such feelings. This finding seems to refute our hypothesis about the putative direct mood-enhancing effects of crying. However, note that the effect on mood was measured immediately after the experimental manipulation, while, as we stated before, the self-soothing effects of crying may need some time to develop. Furthermore, we propose that the awareness of one’s own crying actually may have self-soothing effects, but through more complex cognitive mechanisms than the one of a tactile nature addressed here, that is, through self-image improvements.

***Self-image improvements.*** De Wied et al. (1995) reported a paradoxical relationship between the intensity of empathic distress during film watching and the degree of enjoyment reported afterwards. Much the same as is the case for scary movies, roller coasters, and haunted houses, participants who experienced more (empathic) distress during the film, afterwards indicated that they enjoyed the film as a whole more, compared to those who experienced less distress. Among several other explanations, the authors propose that people may in fact enjoy strong emotions, including sadness or fear if these feelings are not associated with real situations, and are thus experienced solely as bodily arousal rather than as feelings connected with serious negative events. These authors further suggest that experiencing empathy might be reinforcing because it might make people aware that they are really human and are able to form social bonds. The same line of reasoning can be applied to crying. Indeed, Simons et al. (2013) found that attempts to up-regulate crying, that is, to invest effort in increasing the probability of bringing tears to one’s eyes, are often driven by the individual’s aim to increase his/her own negative feelings and to prevent thinking about oneself as indifferent and non-emotional. People thus tend to associate crying with being a warm person and because of that encourage their own crying to maintain a warm and positive self-image. Such a motivation can, of course, be hardly separated from the motivation to manage the image that other people have about an individual, which illustrates the complex interplay between the inter-individual and intra-individual functions of crying. However, the improvements in one’s self-image or the mere perception of an individual that his/her behavior is appreciated by others may both have self-soothing properties. This could be accompanied by the activation of social engagement system and followed by changes in PNS activation and OT levels.

***Achieving a new perspective on or resolution to a sad event.*** Efran and Spangler (1979) presented a two-factor theory, which proposes that crying first appears during an emotionally arousing event in the phase of recovery *after* the initial phase of arousal. The theory posits that the onset of crying indicates that a psychological barrier, which is characterized by frustration, has disappeared or can be given up, which is accompanied by autonomic shifts from arousal to recovery. The authors found support for such an interpretation in a study in which they requested participants to indicate which parts of an exceptionally emotional film elicited their crying. Participants typically reported sadness and frustration when barriers were presented and tearfulness only when barriers disappeared or at the appearance of a solution for the problem. Relatedly, in the study by Bylsma et al. (2008), participants were asked about both the reasons to stop crying and about their mood after a given crying episode. Reasons such as feeling re-stabilized, situation improvement, achieving a goal, changed perception of situation, and finding peace with the situation that caused the crying, were all found to be related to subsequent mood improvements. Similarly, mood improvement after crying was found to be higher if the events that resulted in crying were resolved (Cornelius, 1997; Bylsma et al., 2008) which suggests that relief is more likely to be reported when the negative event has evolved in a positive direction, perhaps through the effects of crying. These findings together suggest that crying might accompany other, e.g., cognitive or social processes that have self-soothing consequences. However, these findings still do not offer an answer whether crying itself does have such properties.

***The role of learning.*** According to the learning theory, behavior that has been rewarded in the past becomes automatically associated with the experience of gratification. This association is expected not just in the terms of motivation for repeating the same behavior but also in terms of affective states that may precede the expected gratification (or the escape from punishment). Thus, when an individual has displayed behavior that previously elicited social support and comfort (i.e., social-soothing), which is basically rewarding, (s)he will expect that the same behavior will be followed by similar gratification in the future. In other words, such behaviors might activate brain systems that are related to the expectancy of reward (note the correspondence with the neural systems proposed to be involved in emotional numbness discussed above). Crying behavior, with its strong comfort eliciting effects, may thus also be regarded as a behavior that, when triggered, evokes associations with reward. On the other hand, there is some anecdotal evidence suggesting that individuals who experienced strong aversive reactions to crying, such as physically abused or traumatized individuals (e.g., a child who is hit when crying), may come to see crying as a sign of impending punishment. That is why there might be individual differences in the associations of crying with reward or punishment, and why the tendency to associate one’s own crying with reward may result from previously experienced self-soothing and mood improvement effects.

However, not only operant conditioning, but classical conditioning may play a role as well. In this case, crying (or any expression of distress) may be regarded as the conditioned stimulus, whereas comforting by others may represent the unconditioned stimulus. The conditioned response would thus be a reaction that is otherwise (as unconditioned reaction) elicited by social-soothing, for example increase in OT and an associated enhanced sense of well-being (see Vingerhoets, 2013).

#### Behavioral mediation

Regarding the behavioral mechanisms, self-soothing effects of crying could possibly be attributed to some specific components of the crying process: acoustical utterances, sobbing, production of tears, and any other crying-related behavior. Earlier, we already addressed the role of the production of tears, feedback of facial muscle activity, and sobbing (the inhalation of cold air). In this section we will consider another possible specific role of sobbing. More specifically, we draw a parallel between the repetitive and rhythmical properties of stereotypies and sobbing, and we hypothesize that the same mechanism is responsible for the self-soothing effects of all kinds of stereotyped behaviors and of sobbing. In our view, it is the rhythmical repetition of behaviors that is at the core of that mechanism.

*Stereotypies* are seemingly functionless behavior patterns displayed by humans and animals, characterized by repetitive movements. These behaviors typically occur in stressful situations such as during inescapable fear or frustration. For example, tethered sows may develop some specific stereotypies (e.g., chain-chewing, chomping, and trough-biting) to cope with their chronic stress (Wiepkema and Schouten, 1992). Stereotypies seem to modify arousal and they thus can be regarded as emotion-focused coping responses (Mason, 1991a; Wiepkema and Schouten, 1992). There are indications that the self-soothing effects of these behaviors in animals might result from the release of opioids (see Mason, 1991b).

In humans, stereotypical movements such as body rocking or head bobbing (often observed in mentally retarded and/or autistic children) seem to have the capacity to lower a person’s responsiveness to inner stimuli, including pain (De Lissavoy, 1961), as well as to turn one’s attention away from aversive stimuli (Dantzer, 1986; Willemsen-Swinkels et al., 1998; Gal et al., 2002). Relatedly, psychologically calming effects of practices such as certain yoga mantras and (e.g., Jewish) prayers may also be attributed to their rhythmical properties, which also result in favorable changes in cardiovascular activity (Bernardi et al., 2001). Similarly, decreased heart rate and increased OT levels are related to rhythmical stereotypies such as leg swinging, or various other stereotypic behaviors in children (e.g., Soussignan and Koch, 1985; Willemsen-Swinkels et al., 1998; Hollander et al., 2003). The psychological benefits of rhythm may also explain the positive effects of music on mood (Aldridge, 1994).

Of further relevance is the concept of “interactional synchrony” which stresses the role of rhythms of motion and vocalization that are often alternated and shared by a group of related individuals and which also are characteristic of comforting behaviors (e.g., Gratier, 2003; Kinsbourne, 2006). Such behavioral patterns can be observed in rituals, such as praying, singing, dancing, or even greeting behaviors, which all seem to have distress- and arousal-reducing effects. Anthropologists have also written about the anxiety-reducing and social-bonding promotion effects of singing, praying, or crying together, as it occurred in tribes when being struck by great disasters such as famine, epidemics, or other adversity, or when preparing for war (see Vingerhoets, 2013). And we all know from our own experience that comforting a crying infant also often includes gently rocking, accompanied by singing lullabies. Kinsbourne (2006) proposed a functional link between such synchronized behaviors and the above described stereotypical behaviors in animals and corresponding repetitive behaviors in humans, that both may serve distress-reducing functions.

Interestingly, an infant’s crying bout typically starts with irregular sound patterns. If this fails to result in the desired effect, the next phase of crying is more rhythmical regarding the rise–fall melody, (i.e., the pitch changes), and what is more important to our claims, there is little variation in spacing between successive sounds in this phase. In the further, more prolonged absence of caregiving, crying again becomes arrhythmic (Owings and Zeifman, 2004). This observation of variations in infant crying can be linked to the phases of the attachment system activity, where the *protest crying*, which is more active and intense, is gradually replaced by calmer, sad crying, which has been proposed to be the kind of crying that results in relief (Nelson, 2008). It is thus possible to draw a parallel between a rhythmical type of infant crying, which is proposed to have beneficial effects, and possible self-soothing effects of rhythmical sobbing.

Based on the presented findings pointing to the calming properties of stereotypies and other rhythmical behaviors, especially if they include other individuals, we expect that sobbing may serve similar, self-soothing functions. The possible findings about the shared neural basis of both sobbing and various stereotypic behaviors would further support such a possibility. Unfortunately, research on the neural substrates of sobbing is currently completely non-existent. What we do know is that rhythmical behaviors, that are comparable to sobbing, do produce physiological changes (cardiovascular changes probably reflecting PNS activation, as well as variations in OT and opioids) that are comparable to those that we expect to follow tearful crying. Equally important is the possibility that sobbing is accompanied with the similar cognitive processes to those discussed above.

## CONCLUSION

In this review, we have critically evaluated the available research on the mood-enhancing effects of crying. We have put forward some possible mechanisms, in the physiological, cognitive, and behavioral domain, through which crying may self-soothe. First, it is important to take into account the evidence suggesting that criers most likely report mood improvement if they receive comfort from others. Therefore, any mood benefits experienced (at least a large part of them) may be the result of receiving inter-personal support (i.e. social-soothing). However, these effects may not be considered as self-soothing, which, as we defined it, is expected to produce homeostatic changes or mood increases independent of the elicitation of social support. We thus started from the hypothesis that crying may directly result in returning of mood to baseline levels, that is, without inter-individual mediation. Based on the current evidence, the support for such direct, that is, self-soothing effects of crying is largely inconsistent. We clarified which characteristics (of the individual, the crying antecedent, and the act of crying, as well as reactions of others) jointly determine why only a subgroup of criers experience mood improvement, in particular in crying after controllable situations and when receiving positive reactions from others. The observed inconsistencies may also be the consequence of the previously applied (quasi)experimental designs, for example, because mood was typically measured immediately after (non)crying episodes.

Crucially, to evaluate the possibility that crying has self-soothing properties, there is a need to understand the mechanisms through which these self-soothing effects may occur. We thus identified some alternative putative mechanisms through which crying may directly result in mood increases. More precisely, we proposed that self-soothing effects of crying may share the same physiological, cognitive, and behavioral mechanisms that are responsible for the social-soothing effects of crying. There is converging evidence that such effects of crying (including sobbing and shedding of tears) are mediated primarily through changes in PNS activation, OT, and opioid levels. These changes may or may not accompany and reinforce certain cognitive processes which may result in self-soothing, such as the self-image improvements or the expectancy of comfort/reward, as well as behavioral aspect of crying, that is, sobbing. Future studies would benefit from combination of various behavioral and physiological measures, making multiple mood measurements, as well as from consideration of individual differences in self-soothing effects of crying. Furthermore, due to the current lack of research, the knowledge about the similarities in the effects of sobbing and other forms of crying on subsequent mood would benefit from mere distinguishing between these different forms if crying in the future studies. We are aware that, in addition to the presented evidence, there is currently a great deal of speculation, but we hope that the present contribution will stimulate researchers to design studies which can more adequately evaluate the hypotheses presented here.

## Conflict of Interest Statement

The authors declare that the research was conducted in the absence of any commercial or financial relationships that could be construed as a potential conflict of interest.
